# IMMUND: A Diagnostic and Therapeutic Pipeline to Uncover the Convergence in Functional Perturbation at Early Stages of Neurodegenerative Diseases and Multiple Sclerosis Based on Protein Markers

**DOI:** 10.3390/ijms27125627

**Published:** 2026-06-22

**Authors:** Ashmita Dey, Dwipanjan Sanyal, Krishnananda Chattopadhyay, Ujjwal Maulik, Vladimir N. Uversky, Sagnik Sen

**Affiliations:** 1 Department of Computer Scinece and Engineering, SRM University-AP, Amaravati 522 240, India; 2 Structural Biology and Bioinformatics, CSIR-Indian Institute of Chemical Biology, 4 Raja S.C. Mullick Road, Kolkata 700 032, India; 3 Department of Computer Science and Engineering, Jadavpur University, Kolkata 700 032, India; 4 Department of Molecular Medicine and USF Health Byrd Alzheimer’s Research Institute, Morsani College of Medicine, University of South Florida, Tampa, FL 33612, USA; 5 Molecular Genetics and Genomics Division, New England Biolabs, Ipswich, MA 01938, USA

**Keywords:** neuroinflammation, blood–brain barrier dysfunction, transcriptomics, immune–CNS crosstalk, cell-specific protein markers, druggable targets

## Abstract

Neuroinflammation is a key hallmark of both neurodegenerative and neurospecific autoimmune diseases, including multiple sclerosis (MS), where immune dysregulation contributes to cellular stress, autophagy, and disease progression in Alzheimer’s disease (AD), Parkinson’s disease (PD), and MS. Emerging evidence suggests a shared mechanism behind MS, AD, and PD, driven by chronic interaction between the peripheral immune system and the central nervous system (CNS). While MS was traditionally viewed as a primary autoimmune condition, recent research indicated that all three disorders involve a breakdown of the blood–brain barrier (BBB). This structural failure enables peripheral immune cells and cytokines to enter the brain, causing sustained neuroinflammation and accelerating disease progression. Here, we propose an end-to-end framework for identification of the diagnostic and therapeutic cell-specific protein markers commonly regulated in mild–moderate AD (MMAD), early-stage PD (ESPD), and MS within peripheral blood mononuclear cells (PBMCs). PBMC markers were first identified based on shared differential protein expression, followed by filtering for BBB permeability. Subsequently, sorted cell markers were mapped to disease-specific neural cell types. Our analysis suggests that PBMC-derived cells, including astrocyte- and monocyte-like populations, share overlapping transcriptional signatures and functional similarity with macrophages and neuroglial cells, indicating potential transcriptional similarity or functional convergence. Furthermore, intra- and inter-cellular pathway analysis suggested both shared and disease-specific signaling mechanisms, with kinase–integrin interactions emerging as key regulatory factors. Selected potential seed markers, primarily kinases and immunoglobulins, were further analyzed through evolutionary sequence–structure space to identify druggable structural features. Next, protein moonlighting possibilities were tested to enhance the temporal functional trajectory of the markers for precise therapeutic impact. Hence, the framework provides a robust strategy to identify immune-based disease-specificcandidate diagnostic andpotential therapeutic targets.

## 1. Introduction

Immune dysregulation, which refers to the breakdown of molecular control of immune system processes, is shown to be involved in neurodegenerative diseases (NDs), particularly through the dysfunction of glial cells in the central nervous system (CNS) [1]. Microglial cells, a specialized population of macrophages in the CNS, exhibit a stable and inactive immunophenotype in healthy brains and regulate synaptic plasticity under steady-state conditions [2]. However, the propagation of NDs through an active immunophenotype has been suggested in numerous studies [3,4]. For example, microglial activation initiates complex immune responses by increasing the expression of pro-inflammatory cytokines and Toll-like receptors (TLRs) [5]. A strong inflammatory response can be further propagated through adaptive immune cells during pathogen invasion or chronic injury [6], where blood-derived immune cells such as T and B cells play a significant role [7]. Among these, peripheral blood mononuclear cells (PBMCs) represent a critical circulating immune population capable of infiltrating the CNS by crossing the blood–brain barrier (BBB), thereby linking systemic immune dysregulation to neuroinflammatory processes. Activation of the adaptive immune response is also associated with autoimmune diseases, such as multiple sclerosis (MS) [8]. Similarly, strong neuroinflammatory signals can be triggered by the deposition of misfolded proteins in NDs [9].

Although the relationship between immune responses and NDs is not entirely straightforward, the involvement of immune responses has been consistently reported in the literature across disease stages. Several independent studies have documented elevated levels of pro-inflammatory cytokines, including IL-1β, IL-6, and TNF-α, in both early and advanced stages of AD, PD, and MS. Furthermore, immune-related genetic risk loci identified through genome-wide association studies (GWAS) have been reported to be shared across these diseases, implicating innate immune pathways in disease susceptibility. These immune alterations have been shown to correlate with the severity of neuronal loss, cognitive decline, and motor dysfunction, suggesting that sustained neuroinflammation actively contributes to disease progression rather than being merely a secondary consequence of neurodegeneration. Collectively, these observations highlight peripheral immune cells, particularly PBMCs, as accessible and informative indicators of CNS immune dysregulation, motivating blood-based biomarker discovery approaches such as the one proposed in this study [10]. For instance, misfolded amyloid-beta (Aβ), one of the primary pathological factors in Alzheimer’s disease (AD) [11], contributes to the dysfunctional activation of microglial cells by interacting with cytokines, receptors, and pro-inflammatory mediators [12]. Microglia are also associated with the phagocytosis of extracellular Aβ, its degradation, and the formation of Aβ fibrils [13]. In addition, systemic infections have been reported to increase the risk of AD by elevating the expression of TLRs [14]. Importantly, inflammatory alterations have already been observed during the mild cognitive impairment (MCI) stage [15]. However, after the early phase of microglial activation, it becomes difficult for microglial cells to sustain strong inflammatory or immune-mediated fatal signaling [16]. Therefore, the transition stage from MCI to AD represents a critical window for early diagnosis of the disease [17]. Parkinson’s disease (PD), the second most common aging-related ND, is characterized by the deposition of misfolded α-synuclein in dopaminergic neurons within the substantia nigra (SN) [18]. Pathological findings have indicated that PD is characterized by analogous patterns of microglial activation and chronic neuroinflammation [19]. In such cases, elevated levels of TLRs, interleukins (ILs), and chemokines have been observed in patients compared with healthy individuals [20,21]. These findings collectively support the notion that innate immunity contributes significantly to the pathogenesis of NDs. Although effective therapeutic interventions remain limited, several anti-inflammatory drugs have been proposed to delay the progression of AD [22].

**Figure 1:**
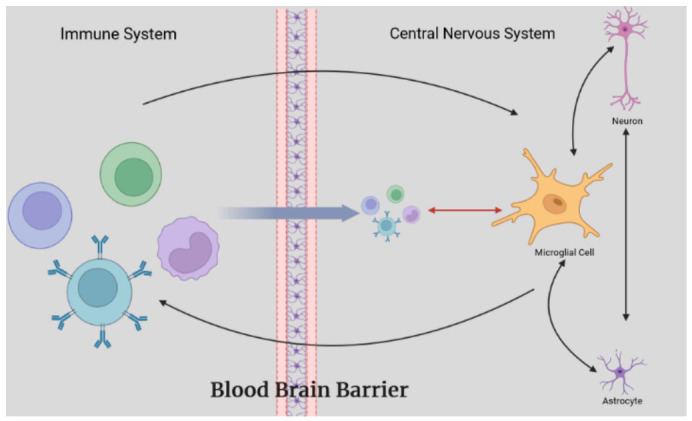
This schematic diagram illustrates the immune crosstalk between the peripheral system (PS) and CNS. In the PS, resident peripheral immune cells—including T lymphocytes (purple), monocytes (blue), B cells (green), and NK cells (teal)—can become activated and infiltrate along peripheral motor nerves and neuromuscular junctions in response to inflammation mediators derived from microglia. In the CNS, microglia, the resident immune cells, can become activated and release either pro-inflammatory (e.g., IL-1β, IL-6, TNF-α) or anti-inflammatory substances, and interact with infiltrated peripheral immune cells. The activation, migration, and proliferation of microglia are controlled by astrocytes. Dysfunction of CNS barriers, such as the BBB and blood–spinal cord barrier (BSCB), can facilitate the infiltration of peripheral immune cells and accelerate harmful interactions. Consequently, inflammatory responses propagate across both systems, leading to motor neuron (MN) death, injury to MN axons, and dysfunction of neuromuscular junctions.

Interestingly, antibodies, cytokines (including *IL-1β*, *IL-6*, *TNF-α*, *IL-12*, and *TGF-β*), and aberrantly acting kinases (such as PAK1, CDK12, and PHKG2) have been identified as potential biomarkers for NDs and autoimmune diseases such as MS [23], particularly during the early stages of these pathologies. Therefore, these molecules could serve as promising candidates for early diagnosis and potential therapeutic intervention [24]. In many NDs, misfolded and aggregated proteins interact with neuroglial components [25]. For example, activated microglia bind to Aβ and tau proteins through receptor-mediated and cytokine-driven mechanisms involving TLRs, tumor necrosis factors (*TNFs*), and related mediators. These immune-mediated interactions are strongly associated with the loss of cognitive function. In contrast, MS is primarily an autoimmune disease in which antibody-mediated responses play a central role. ND and autoimmune diseases are characterized by disease-specific immune cell activation profiles, in which the cellular specificity of immune responses plays a vital role in shaping the path of the disease. Despite growing interest in neuroinflammatory mechanisms, no prior study has jointly explored both common immune functional signatures and condition-specific variations among AD, PD, and MS through a unified multi-omics approach, to the best of our knowledge. The early stages of ND can also involve peripheral immune cells capable of crossing the BBB, such as PBMCs. Other NDs and autoimmune diseases are characterized by complex, immune-driven processes that involve specific cellular cohorts, where immune cell specificity plays a critical role in pathogenesis, tissue destruction, and disease progression. However, a systematic, unified approach to compare these specific aspects across many diseases is still lacking, as research often fails to concurrently analyze generic functional similarities alongside distinct disease-specific dissimilarities. The conceptual framework in Figure 1 delineates the gap between experimentally validated hypotheses and the system-level impact of immune biomarkers on neuro-specific perturbations and disease progression.

In this study, we aim to investigate cell-type-specific mechanisms associated with the immune responses and neurodegenerative diseases. Given that AD, PD, and MS share overlapping yet distinct neuroinflammatory underpinnings—spanning innate microglial dysfunction, peripheral immune infiltration, and adaptive immune dysregulation—we selected early-stage PD (ESPD), mild–moderate AD (MMAD), and MS as our primary disease conditions. The study was initiated by identifying differentially expressed proteins commonly associated with these three conditions, where MCI was used as a reference-controlled stage representing early-phase neuroinflammation prior to overt neurodegeneration, thereby providing a biologically relevant baseline for the comparative analysis. Subsequently, the selected biomarker enrichment profiles were used to identify primary lists of cellular subtypes from PBMC datasets, which were further segregated based on their potential ability to cross the BBB. These cell types were then used to examine their distinct contributions to the three diseases by integrating single-cell RNA sequencing datasets for each condition. For each cell type, we aimed to uncover mechanisms of disease progression and inter-dependencies that could assist in selecting primary protein families with substantial impact on disease pathology. Finally, the selected proteins were subjected to structural stability analysis, where evolutionarily conserved structural cores were utilized for docking analyses to explore potential therapeutic strategies.

## 2. Results

### 2.1. Identifying Differentially Expressed Proteins

**Figure 2:**
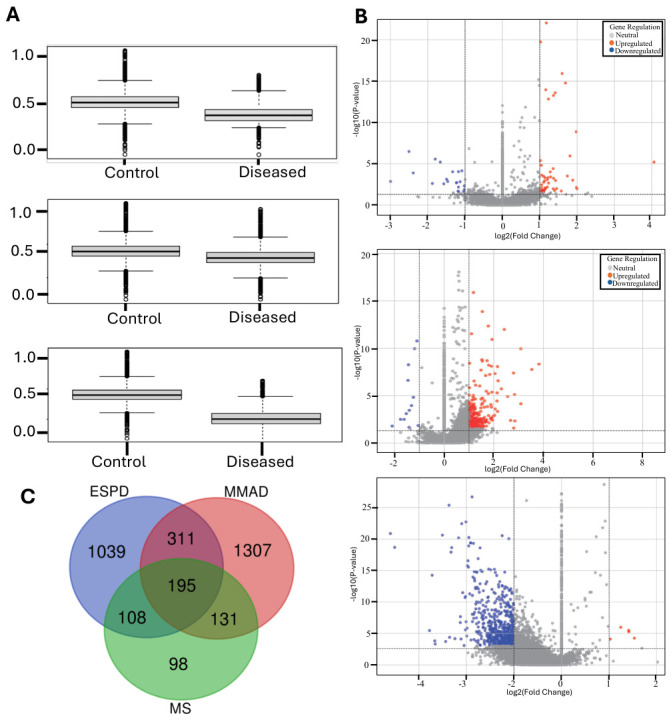
The box plot in (**A**) represents the expression rate in the both control and diseased group. (**B**) The volcano plot is performed to show the up/downregulated proteins in each diseased dataset. (**C**) The Venn diagram is performed by considering the differentially expressed proteins of each of the three diseases. We got 195 common proteins among which two were hypothetic protein; therefore, 177 proteins were considered for further study.

The dataset contains 9481 expressed proteins for each disease. We refined the dataset based on the *t*-test score. Depending on the *t*-test score, the proteins are further considered for identifying the differentially expressed proteins shown in Figure 2A. In Figure 2B, a volcano plot of the distribution of differentially expressed genes between the diseased and MCI groups is shown (reported in Supplementary Table S1). The red and blue dots represent the significantly upregulated and downregulated proteins respectively. The proteins with insignificant differences in regulation are represented as gray dots. The Venn diagram in Figure 2C depicts that 195 autoantigens are shared among the three diseases. Due to the presence of two hypothetic proteins and 16 other unknown proteins, we discarded those autoantigens and considered 177 proteins for further analysis, reported in Supplementary Table S2 along with their uniport IDs.

### 2.2. Understanding the Role of Peripheral Immune Cell Types on Neurodegenerative Disease Progression

**Figure 3:**
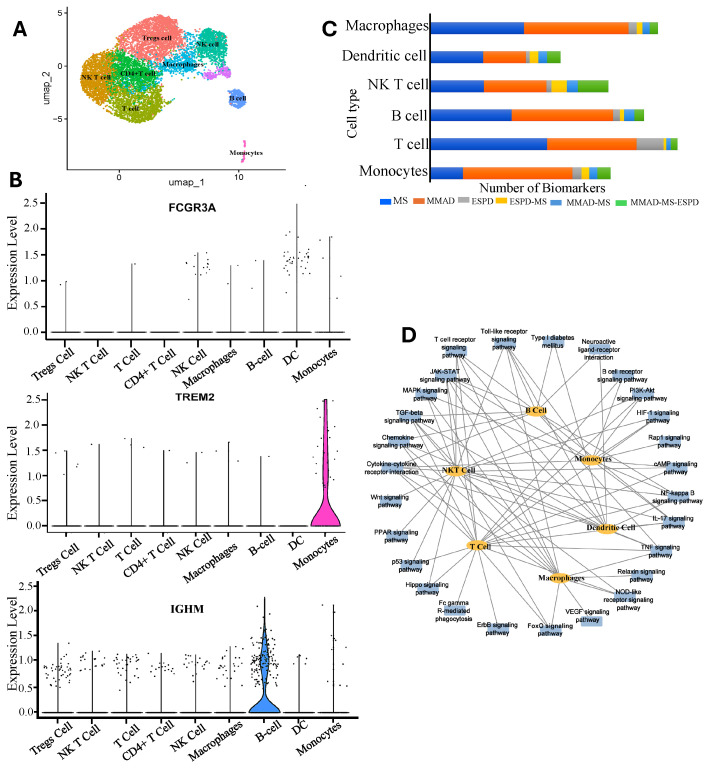
(**A**) To understand the impact of immune cells on neuroinflammation and the progression of neurodegenerative diseases, we analyzed PBMC scRNA-Seq data. (**B**) The expression of a subset of genes among the 177 selected was visualized using violin plots. (**C**) Subsequently, we identified cell types that exhibited the highest number of commonly expressed proteins, as shown in the bar plot in panel. (**D**) These selected cell types were further used to construct a network illustrating the pathways associated with cell types that play significant roles in neurodegenerative diseases.

**Figure 4:**
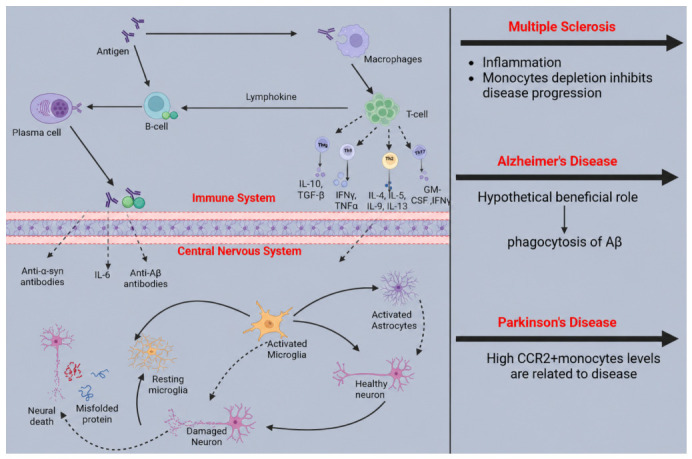
Crucial role of peripheral immune cells in the pathogenesis of neurodegenerative diseases. After antigen stimulation, macrophages and B cells initiate an immune response. Macrophages engulf and present antigens to T cells, which release cytokines such as IL-4, IL-6, IL-12, and TGF-β to regulate the development of Th2, Treg, Th1, and Th17 cells, respectively. These cells, in turn, secrete anti-inflammatory or pro-inflammatory factors that affect neuronal survival. In addition, B cells can be directly stimulated by antigens, leading to the production of pro-inflammatory factors that can enter the brain along blood vessels and contribute to neurodegeneration. Activated T cells can also secrete lymphokines that activate B cells. These activated B cells can then proliferate and differentiate into plasma cells, which produce cytokines and antibodies, including anti-Aβ or anti-α-synuclein antibodies. These antibodies can cross the blood–brain barrier and enter the brain, ultimately reducing neuronal degeneration.

It is recognized now that a timely and well-controlled peripheral inflammatory reaction is verified to be essential for neuroprotection, whereas chronic or uncontrolled peripheral immune reaction is linked to the pathogenesis of neurodegenerative diseases. This “double-edged sword” nature of the immune system in neurobiology indicates that the effect of the peripheral inflammation on the CNS is closely related to the activation of immune cells in peripheral blood, where the peripheral immune system can both support and sabotage the CNS. To understand the impact of peripheral cell types on these three neurodegenerative diseases (AD, PD, and MS), we observed the cell-specific involvement of the immune system towards these neurological disorders. In this regard, publicly available PBMC scRNAseq are analyzed and clustered based on their cell types (as shown in Figure 3A). Furthermore, a bar plot identifies the immune cell types possessing the maximum number of the markers among the selected 177 autoantigens of the three selected diseases. Figure 3B represents six cell types along with their disease’s markers verified from the literature. Additionally, the role of six immune cells in the neuroinflammation and progression of neurodegenerative diseases are explained based on their functionality. In Figure 4, the role of immune cells in the neuro disorder is explained based on our findings. Through a network representation shown in Figure 3C, we have depicted that, after encountering antigens, immune cells such as macrophages and B cells kick-start an immune response [26]. Macrophages engulf and display antigens to T cells, which then release cytokines, such as IL-4, IL-6, IL-12, and TGF-β to regulate the development of various types of T cells, including Th2, Treg, Th1, and Th17 cells [27]. These different types of T cells subsequently produce anti-inflammatory or pro-inflammatory factors that can impact neuronal survival. Furthermore, B cells can be directly activated by antigens, which trigger the production of pro-inflammatory factors that can enter the brain via blood vessels and contribute to neurodegeneration. Activated T cells can also secrete lymphokines that activate B cells [28]. These activated B cells can then multiply and differentiate into plasma cells, which can generate cytokines and antibodies, including anti-Aβ or anti-α-synuclein antibodies. Ultimately, these antibodies can traverse the BBB and enter the brain, helping to mitigate neuronal degeneration. In Supplementary Table S3 we report the effect of immune cells in the pathogenesis of the selected neurodegenerative diseases in detail.

### 2.3. Deciphering How Peripherally Expressed Genes Change Their Functionality in Glial Cells

**Figure 5:**
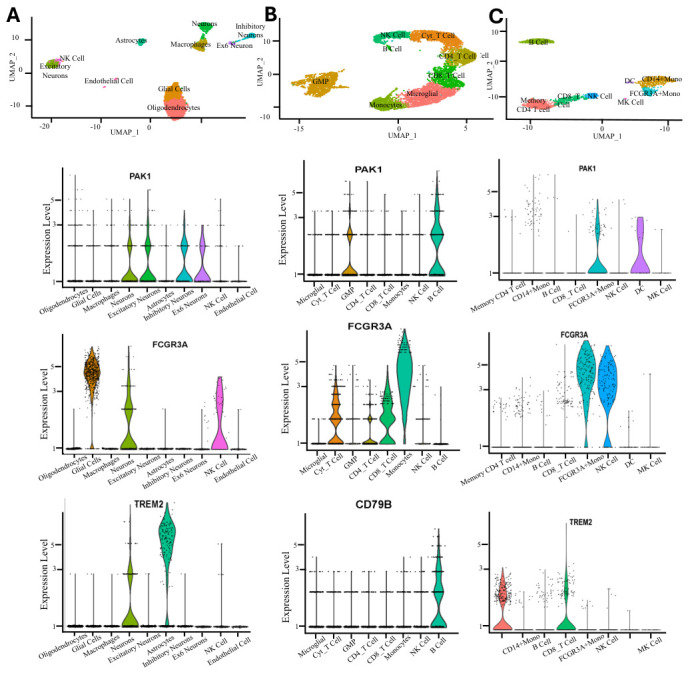
(**A**–**C**) represent proteins from AD, PD, and MS, where violin plots represent few proteins and their cell specific enrichment using violin plots respectively. This figure illustrates that single-cell data specific to each of the three diseases was curated, and it displays the cell-type-specific expression of proteins from the 177 selected proteins of each family, as reported in Supplementary Table S1.

PBMCs show early immune activation signatures. Similarly, brain-specific single-cell RNA-seq analysis suggested glial activation, neuronal loss, or microglial inflammation. In this regard, we conducted analysis of datasets from AD, PD, and MS. The goal was to explore gene expression across different cell types and uncover molecular potential mechanistic associations contributing to disease progression. The disease-specific UMAP and the violin plot of the genes showing noticeable impact in the PBMC dataset are shown in Figure 5. The figure depicts that PAK1 (p21-activated kinase 1, also known as serine/threonine-protein kinase PAK1) is highly expressed in neurons in AD, whereas it exhibits significant expression levels in B cells and monocytes in PD and in glial cells in MS. *FCGR3A* (Fc-gamma RIII-alpha, also known as low-affinity immunoglobulin gamma Fc region receptor III-A) is predominantly expressed in oligodendrocytes, natural killer (NK) cells, and astrocytes, while *CD79B* (B-cell antigen receptor complex-associated protein beta chain) and immunoglobulin genes showed high expression in B cells and neurons. Furthermore, we have reported the expression of another important gene TREM2 among the three diseases.

The cell-type-specific functionality of each disease is shown using a heat-map in Supplementary Figure S1, where the cell types that possess high levels of selected proteins are considered and reported. We have noticed that there are some common pathways among all the disease datasets. Even if the gene set is different in each list, they still showed some common pathways thereby revealing the functional similarity among the neurodegenerative diseases. For further understanding, a disease-specific pathway semantic similarity network was established to unveil the crosstalk between the cell types. In AD, macrophages showed a high score with astrocytes, whereas macrophages were highly connected with microglia cell types in PD. On the other hand, in the MS environment, microglia show connection with CD4 T cells, as illustrated in Supplementary Figure S2.

### 2.4. Sequence–Structure Space Analysis of the Selected Autoantibodies

Building upon the transcriptomic findings, we next sought to explore the molecular features and evolutionary patterns of the identified candidate genes and their protein products. To this end, we performed sequence–structure space analysis on the top-ranked proteins derived from the cell-type-specific expression data. This allowed us to assess domain-level classification, intrinsic disorder, and binding versatility factors that could further explain the functional adaptability of these proteins across cell types under neurodegenerative conditions. The fasta sequencess of the proteins are given in Supplementary Materials.

#### 2.4.1. Sequence Space Analyses

The top ten selected proteins are found distributed in three families: Viz., immunoglobulin C1-set domain (Pfam ID: PF07654), immunoglobulin V-set domain (Pfam ID: PF07686), and protein kinase (Pfam ID: PF00069). Among them, a total of three members (UniProt IDs: P55899, P01871, and P0DOY3) with multiple *p*-value occurrences belonged to two families, i.e., PF07654 and PF07686. From the initial Shannon Entropy (SE) scoring, we found that these two families have higher levels of intrinsic disorder. In addition, these families showed multiple binding partners or multiple binding propensities with the same partner (defined by the term multi-occurrences). However, in the case of the third family (Pfam ID: PF00069) the disorder trait was found to be lower (as the threshold is 2.9). Interestingly, the proteins with higher values of disorder trait were found to belong to the immunoglobulin family proteins (PF07654 and PF07686). We then identified that six out of the ten proteins were immunoglobulins, while the other four autoantigens were kinases (belonging to PF00069).

**Figure 6:**
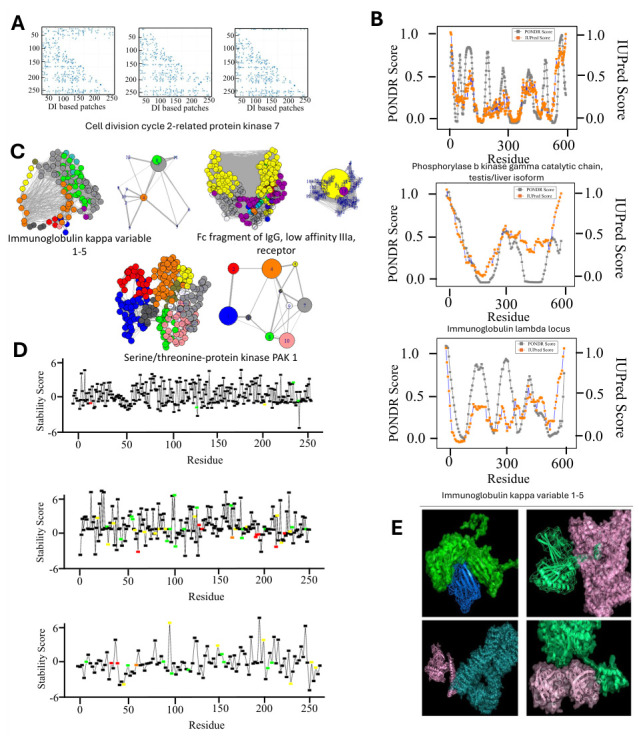
Comparative analysis of protein structural stability, disorder propensity, and interaction patches across selected immune-related proteins. (**A**) DI-based patch analysis (middle panels) identifies regions likely involved in molecular interactions. (**B**) Disorder prediction profiles generated using PONDR and IUPred highlight intrinsically disordered regions, indicating potential functional flexibility. (**C**) The community cluster modules of protein structures are performed after performing modularity detection. (**D**) Residue-wise stability scores illustrate fluctuations in structural stability along the protein sequences. (**E**) The docking validation study and corresponding docking. Together, these integrated analyses provide insights into the structural dynamics and functional relevance of immune-associated proteins in disease contexts.

Then, using the sequence information of the aforementioned three protein families, we performed multiple sequence alignment (MSA) followed by direct coupling analysis (DCA) study (Figure 6A), with details shown in Supplementary Figure S3. Through the course of evolution, the impact of substitution of a residue at a particular site would be nullified by the alteration at another position in proximity in the 3D protein structure. These two positions are defined as evolutionary coupled/co-varying pairs. DCA study helped us identify such coupled pairs, which played a key role in better understanding the important regions in the protein structures. Hence, coupling propensities at intrinsically disordered regions (IDRs) have been computed.

The intrinsic disorder propensities of individual proteins were evaluated using two prediction models, namely PONDR-VLXT and IUPred. The regions found to be common using both algorithms were considered to be the effective disordered regions (Figure 6B). The rest of the proteins are shown in Supplementary Figure S4. The outcome complied with the SE score obtained for the three families. The predicted intrinsic disorder scores were mostly justified by the higher SE scores for two families (PF07686 and PF07654) and by the lower SE score of P0DOY3 family proteins. Similarly, IDRs within the evolutionary coupled patches helped to explore the distribution of intrinsic disorder predisposition. More elaborately, seven out of 10 proteins had disordered regions, which were found to exist within the evolutionary co-varying patches shown in Figure 6D. Other examples are shown in Supplementary Figure S5 due to extremely low coupling propensities. Among the seven proteins, the PF00069 family has four members, the PF07654 family has one member, and finally, the PF07686 family has two members in the list. It can be suggested that the IDRs with high co-varying propensity would have significant impact on the overall structure of the proteins. We infer that such effective disorderedness can partially enforce the creation of the globule-like structure at the monomeric stage that justifies the presence of the maximum number of immunoglobulin families in the list of proteins with high disorder.

The protein members from the PF00069 family were found to contain many co-varying patches that were distributed mainly within a particular stretch or region. These have been termed in this article as locally distributed co-varying patches (see in Figure 6B). Among them, protein immunoglobulin lambda constant 3 (IGLC3, Uniprot ID: P0DOY3) was found to have two disordered regions, one from two to 15 (positions from two to 7 were considered to be effective disordered regions) and another from 48 to 58 (48–54 and 57 were in the effective disordered regions). For the first disordered range, 24 unique residues were predicted to be coupling pairs with different direct information (DI) scores (Supplementary Table S4). In the second disordered range, 12 different amino acid positions were observed to be co-varying. Similarly, protein immunoglobulin kappa variable 1–5 (IGKV1-5, UniProt ID: P01602) was found to contain mainly one effective disordered region (sequence 1–8). Interestingly, the amino acid sites within these ranges were observed to couple with 35 different positions throughout the sequence. Similarly, proteins phosphorylase b kinase gamma catalytic chain, liver/testis isoform (PHKG2; UniProt ID: P15735), serine/threonine-protein kinase PAK 1 (UniProt ID: Q13153), and immunoglobulin lambda variable 2–14 (IGLV2-14; UniProt ID: P01704) had 21 and 17 coupled pairs that were distributed mainly in the ordered regions. Here, it was observed that the positions in the proteins that belong to the families with lower disorder traits (having low SE scores) mainly had coupled pairs that showed the propensity to be ordered. The aforementioned analyses provided clear insight regarding the evolutionary conservation, co-variation as well as individual sequential orchestrations. However, the observations can be further stringent through the structure-based analysis.

#### 2.4.2. Structure Space Analysis

The structure space analysis has two levels: I. structure network analysis, and II. alanine mutagenesis. Here we had compared and merged the sequence space observations with detailed information obtained from the structure network study. The PDB structures of the selected protein chains that were associated with the disease systems were chosen for this study [29]. The selected PDB structures were then subjected to structure network study. Structure network analyses provided an understanding of the internal arrangement and inter-dependency of the amino acid residues in protein structures. In this analysis, all residue networks of the proteins were generated that were further split into interlinked community cluster networks by means of Girvan–Newman algorithm. In these cluster networks, the highly interacting amino acid residues were assembled in the residual communities as shown in Supplementary Figure S5. Using betweenness centrality calculations, the influence of a particular residue on the overall structural dynamics was then explored by considering its fluctuation profile. The structures with maximum disorderness were found to lack a diverse set of communities based on the residue–residue interaction. In terms of color modules, residues were flocked together in a minimum number of modules for five out of 10 proteins (UniProt ID:- Q13188, P04440, P55899, P01871, and P0DOY3). Among the above mentioned five proteins, the last three multi-occurred, while the other five proteins (UniProt IDs:-P15735, Q13153, Q9NYV4, P01704, and P01602) have six, 10, 10, 10 and 12 modules respectively. Therefore, the maximum number of proteins could not assemble in ordered structures. Subsequently, the concept of alanine mutagenesis was introduced to interpret the contribution of a single residue towards the stability of a protein by means of the stability index value. As alanine is a non-bulky amino acid, the mutagenesis at every residue can provide the rate of stability. This approach was used for all the selected autoantigens (Figure 6D). One protein is shown in the figure as a representative of the families and other proteins are reported in the Supplementary Figure S6.

The quality assessments of the selected proteins were performed, and the respective non-bonding clashes were calculated. By applying quality assessment, the effect of Van der Waals interactions (VdW), torsional angle, etc., were calculated on each residue (marked in different colors), and the changes in energy due to non-bonding interactions were determined as well (Figure 6D). The H-bond clashes were calculated in the two following ways, i.e., VdW clashes and changes in torsional clashes in the residues. Following the alanine scanning results, comparative rates of fluctuations were given in the color dots. In the case of four multi-occurring proteins, the VdW clashes and the changes in the torsional clashes were observed at all the disordered regions. In immunoglobulin heavy constant mu (IGHM (Uniprot ID:-P01871), VdW clash was observed at residue 200 (consisting of TYR) whereas at residue position 201, photo-isomeric changes in dihedral angles were observed in terms of ω angle. Similarly, IGLC3 (UniProt ID: P0DOY3) was found to have three VdW clashes at residue position seven, 11 and 14 (consisting of residues SER, ALA, and GLN respectively) in the first disordered region whereas the second disordered region had two VdW clashes and one torsional clash at residue position 49, 50 and 52 respectively (residue PHE, ALA, and THR). At residues 39, 26 and 61 (consisting of LEU, ASN, and ARG respectively) of P0DOY3, one Vdw clash and two torsional clashes were observed. On the other hand, only Q9NYV4 has one VdW clash at residue position 124 (consisting of GLN) and coupled partner residue position 77 (consisting of ASP). However, VdW clashes are observed at coupled partners of Q81WQ3, and Q13188 at residue position 204, 69 and 129 (consisting of MET, TYR and, ARG respectively) respectively. Subsequently, P01602 had one Vdw clash at residue position five (a PRO residue). In case of all these three proteins, extreme changes in χ angles at a nearer position of the Vdw clashes were observed, except P55899, in which changes in ω angle had been observed. Using the steric clashes as computed above, we defined the clash zones at the disordered regions, which would be important patches/regions for binding interactions. In addition, based on the sequence space results, the coupled pairs were also considered to be binding facets for the proteins. Using the above criteria, the non-covalent interactions at the coupled partners of the disordered regions were explored.

### 2.5. Expression Validation of Selected Protein Markers Using Independent Public Datasets

To address the absence of experimental validation inherent to a computational framework, we performed an independent cross-validation of the selected seed markers using three publicly available datasets corresponding to AD [30], PD [31], and MS [32] patients. Expression levels of the identified markers were compared between disease and control samples using Welch’s two-sample *t*-test, and the resulting *p*-values were adjusted for multiple hypothesis testing using the Benjamini–Hochberg false discovery rate (FDR) correction. The distribution of expression values for each marker is illustrated in Supplementary Figure S7.

The statistical analysis revealed significant differential expression for multiple candidate markers across the three independent cohorts. In the AD dataset, *PAK1* ($p=4.04\times 10^{-10}$), *TREM2* ($p=6.54\times 10^{-6}$), *FCGRT* ($p=3.20\times 10^{-8}$), *CDK12* ($p=1.14\times 10^{-5}$), *CD79B* (p=0.0022), and *STK3* (p=0.0022) exhibited significant differences between disease and control samples. In the PD dataset, *FCGR3A* (p=0.0179) and *STK3* (p=0.0349) were significantly dysregulated. Similarly, in the MS dataset, *TREM2* (p=0.0176) and *STK3* (p<0.0001) showed significant differential expression between disease and control groups.

Among the identified markers, *STK3* demonstrated consistent and statistically significant dysregulation across all three neurodegenerative disease cohorts, while *TREM2* was significantly altered in both AD and MS datasets. The reproducibility of these findings across independent datasets supports the robustness of the IMMUND framework and provides additional evidence for the biological relevance of the identified seed markers. Furthermore, the consistent disease-associated expression changes observed for several kinases and immunoglobulin-related proteins suggest their potential utility as candidate therapeutic targets and warrant further experimental investigation.

### 2.6. Docking Validation

To validate the identified binding facets, docking study was performed with the interaction partners selected from the PPI network. From protein–protein docking study, we found that (PDB ID:1LDS) was housed in the binding groove of protein IgG receptor FcRn large subunit p51 (FCGRT, UniProt ID:P55899). This interaction mainly involved the residue stretch from 48 to 56 of 1LDS. Since serum albumin (ALB) is also known to be a binding partner of P55899, we used docking study to find out their interaction domains. The binding of ALB was found to occur through the terminal loop region from residue seven to 41 of P55899. We observed that P55899 used two binding regions, which are opposite to one another, to interact with these two binding partners (1LDS and ALB). The most favorable bound conformation of serine/threonine-protein phosphatase 2A (PP2A; PDB ID:2IAE) and serine/threonine-protein kinase 3 (STK3, UniProt ID: Q13188) indicated that they were in proximity by involving 106 atoms. The terminal helix and loop regions of Q13188 were involved in this interaction, as shown in Figure 6E.

## 3. Discussion

In this study, we analyzed disease-specific autoimmune blood-based biomarkers of early PD, mild–moderate AD, and MS at the mild cognitive impairment (MCI) stage to investigate the relationships among these three diseases. Our approach included: (1) comparison of gene expression changes using volcano plots, and (2) single-cell analysis to evaluate the contribution of immune cell types to neurodegenerative disease progression. Our findings suggested both shared and distinct gene expression patterns across the three diseases, with strong cell-type specificity.

Therefore, our comparative analysis captures the common and distinct molecular profiles associated with early stages of these NDs. Neuroinflammation represents a key mechanistic commonality among AD, PD, and MS, as previously reported [33]. Recent studies have shown that cytokines, such as *IL-1β*, *IL-6*, and *TNF-alpha*, are commonly dysregulated across these diseases [34], consistent with the 177 shared biomarkers identified in our protein array analysis. We further investigated the progressive modulation of neuro-markers in relation to peripheral immune responses, BBB dysregulation, and neurospecific cellular activity. Our results indicate that PBMC cell types—particularly B cells, monocytes, and dendritic cells—are significantly enriched among the shared biomarkers, suggesting a potential role for the immune–neurological axis in early disease progression.

Among immune cell populations, peripheral macrophages exhibited the highest similarity in gene expression changes across MS, AD, and PD. Previous studies have shown that AD and immune-mediated disorders share common genetic risk variants, and that AD- and MS-associated genes are enriched in peripheral macrophage gene signatures [35,36]. Our observation of commonly upregulated macrophage genes in MS and AD supports the possibility that these diseases share macrophage-driven molecular mechanisms. However, it remains unclear whether macrophage activation is a primary driver or a secondary consequence of neuronal and synaptic damage in AD and MS. Although several studies suggested an early causative role for macrophages [37], further investigation is required to establish causality. While upregulated macrophage genes may represent potential therapeutic targets for early-stage intervention, the beneficial roles of macrophage activation and potential adverse effects must also be carefully considered before clinical translation.

B cells have also been extensively studied in neurodegenerative diseases. As key components of adaptive immunity, B cells participate in antigen presentation, cytokine secretion, and immune regulation [38,39]. In particular, autoreactive B cells play a critical role in autoimmune neurological disorders. These cells produce pro-inflammatory cytokines, such as *IL-6*, *TNF-α*, and *GM-CSF*, while also secreting anti-inflammatory mediators including *IL-10* and *IL-35* [40,41]. Age-associated decline in B-cell populations contributes to immunosenescence and may influence the pathological progression of neurodegenerative diseases. Reduced levels of peripheral B-cell subsets have been reported in AD patients, potentially associated with genetic alterations in these cells [42]. In PD, B cells are rarely detected in the brain, although IgG deposition is observed in dopaminergic neurons and Lewy bodies [39,43]. In MS, B cells are present in CNS lesions across disease stages and display a pro-inflammatory cytokine profile [44]. Notably, B cells from HCMV- MS patients responding to HCMV-encoded antigens show an enhanced pro-inflammatory response compared with HCMV+ MS cases, suggesting that persistent HCMV infection may attenuate B-cell inflammatory responses [45,46]. These findings highlight the therapeutic potential of B-cell-directed strategies in MS.

Autoreactive B cells can differentiate into plasma cells that produce antibodies targeting myelin and oligodendrocyte proteins, leading to tissue damage through complement activation and recruitment of immune effector cells such as NK cells [47]. In neurodegenerative patients, these cells often exhibit increased production of pro-inflammatory cytokines and reduced secretion of protective cytokines compared with healthy individuals. In addition, they may form ectopic lymphoid structures in the meninges containing activated B cells and follicular dendritic cells, sustaining T-cell activation and promoting chronic CNS inflammation [48]. Adaptive immune cells, particularly T lymphocytes, have also been implicated in neurodegeneration in PD [27]. Our results support this notion. The regulatory patterns of T-cell-associated genes, including IL24, STAT6, APOE, and APP, validate our analytical framework and provide proof-of-principle evidence for identifying shared disease mechanisms. Activation of adaptive immune responses is therefore not restricted to MS but also occurs in classical neurodegenerative disorders, such as AD and PD. An imbalance between pro-inflammatory effector T cells and regulatory T cells (T_*reg*_s) contributes to neuroinflammation and neuronal damage [27]. Consequently, expansion of anti-inflammatory, neuroprotective T_*reg*_ populations represents a promising strategy for slowing disease progression. Targeting peripheral immune cells may further facilitate drug development, as such interventions may not require therapeutic agents to cross the blood–brain barrier. During neuroinflammatory responses, PBMCs exhibit morphological alterations associated with macrophage activation and microglial differentiation [49]. These observations support the hypothesis that cellular relocalization and morphological remodeling contribute to functional modulation of disease processes following BBB traversal. While such phenomena are well documented in MS particularly for T cells, B cells, and monocytes [27], their relevance in MMAD and ESPD remains less clear. Nevertheless, interactions between astrocytes and amyloid-β deposition have been previously described [50]. The cytokines identified in this study may trigger intra-cellular pathways that promote neuroinflammation, whereas dysregulated kinases may induce aberrant phosphorylation events that impair key disease-associated proteins. These findings collectively suggest functional dependencies during the early stages of disease progression.

Astrocytes and neuroglia have previously been implicated in disease-specific pathogenic mechanisms, including neuroglial activation associated with Aβ aggregation, α-synuclein aggregation, and demyelination in AD, PD, and MS, respectively [51]. Astrocytes also play a central role in maintaining BBB endothelial integrity [50]. To further investigate the cellular basis of these biomarkers, we analyzed disease-specific scRNA-seq datasets. Notably, PBMC cell types exhibited shared expression trajectories, particularly between macrophages and astrocytes and between monocytes and neuroglial cells, suggesting coordinated phenotypic and morphological adaptations.

Additionally, we examine whether inter-cellular crosstalk contributes to disease progression. Integrins identified in our dataset were linked to extracellular matrix (ECM), adhesion, and migration pathways, consistent with pathway–pathway interactions observed between monocytes/T cells and microglial cell types. Dysregulation of these integrins may disrupt cellular communication networks and promote aberrant signaling between immune and neural cells within the CNS. While categorizing the list of selected proteins, we have identified a group of kinases. Now, these kinases are either responsible for intra-cellular signal transduction, phosphorylation, or restricting inter-cellular crosstalk being present as cell surface enzyme. Although aberrant kinase activity is a well-established diagnostic marker in malignancies and autoimmune diseases [52], its role in neurodegenerative disorders remains insufficiently explored. Interestingly, our analysis identified several kinases interacting with integrins. As mentioned earlier, we hypothesized that aberrant phosphorylation in the cell surface barring the integrin activity further affected natural cell-to-cell communication.

Further analysis identified three major protein families as potential therapeutic targets. Sequence–structure space analysis was performed to identify evolutionarily conserved domains involved in ligand binding. Among these families, PF00069 corresponds to a kinase family associated with diverse biological functions. Therefore, we used the MoonProt database to assess potential protein moonlighting activity.

Sequence–structure space analysis further suggested potential binding facets that may serve as drug-targeting regions. The quality assessment analyses represent key structural facets based on sequence- and structure-level information from largely unstructured proteins, including STK3 (UniProt ID: Q13188), HLA class II histocompatibility antigen, DP beta 1 chain (HLA-DPB1; UniProt ID: P04440), FCGRT (UniProt ID: P55899), IGHM (UniProt ID: P01871) and IGLC3 (UniProt ID: P0DOY3). Notably, the structural facets identified for Q13188, P55899, and P0DOY3 were consistent with the regions predicted from non-covalent interaction analyses. For the remaining proteins, structural facets were more difficult to identify due to their structural characteristics. In contrast, these facets were more clearly observed in comparatively structured proteins, such as PAK 1 (UniProt ID: Q13153), cyclin-dependent kinase 12 (CDK12, UniProt ID: Q9NYV4), and IGKV1-5 (UniProt ID: P01602) at module numbers nine, one, three, and two, respectively. Residues involved in non-covalent interactions in the remaining proteins did not exhibit significant disorder. Residue-wise fluctuation information is provided in (Supplementary Table S5). To validate these structural facets, docking analyses were performed with multiple protein partners. The interacting regions between proteins and their respective partners closely matched the predicted binding facets. Notably, one antigen (FCGRT; UniProt ID: P55899) utilized multiple regions to interact with different partners (1LDS and ALB), further supporting the accuracy of our sequence and structure-based predictions.

Functional annotation further strengthened these observations. The moonlighting properties of the analyzed proteins suggested functional associations with multiple diseases. Specifically, diseases such as MMAD, ESPD, and MS appear to share common proteins, although they do not necessarily share the same biological pathways. The presence of extensive intrinsically disordered regions in these proteins likely contributes to their ability to participate in multiple functional contexts. Combined with the observed expression-level variations, these features highlight the potential diagnostic and therapeutic relevance of these proteins. As these proteins were identified as moonlighting proteins (validated using MoonProt), the results of our expression and sequence–structure analyses can be interpreted in the context of multifunctional protein behavior. While four proteins were previously reported in the literature with associated pathways, two proteins, i.e., CDK12 (UniProt ID: Q9NYV4) and IGKV1-5 (UniProt ID: P01602), emerged as potential novel markers in this study.

This study is primarily computational and integrative in nature. Although the identified biomarkers, pathways, and structural features provide biologically meaningful hypotheses, the findings require experimental validation using independent patient cohorts and functional assays. In particular, conclusions regarding therapeutic applicability, causal disease mechanisms, and potential trans-differentiation should be interpreted cautiously until validated through in vitro or in vivo studies. Therefore, the proposed framework should be considered as a hypothesis-generating and prioritization pipeline for identifying candidate biomarkers and molecular interactions associated with neuroinflammatory mechanisms across MMAD, ESPD, and MS.

## 4. Methods

### 4.1. Data Description

The protein expression data of three neuro diseases such as Alzheimer’s, Parkinson’s and multiple sclerosis are collected from the GSE74763 dataset [53]. The dataset contains disease-specific autoimmune blood-based biomarkers of early AD, mild–moderate PD and MS at the mild cognitive impairment (MCI) stage. The authors of the study considered the sera, obtained from a pre-defined age and gender-matched threshold value, of control samples. The 25 ADNI MCI samples are compared with 25 control samples from 9486 human protein microarrays using Prospector (v5.2.3) analysis software. MCI was selected as a biologically relevant intermediate reference condition because it represents an early neuroinflammatory and pre-neurodegenerative state prior to severe cognitive decline. Using MCI as a reference enabled the identification of molecular signatures associated with progression toward MMAD, ESPD, and MS while minimizing late-stage disease-specific confounding effects. The objective was not to treat MCI as a universal healthy control, but rather as an early-stage neuroinflammatory baseline for comparative analysis.

**Figure 7:**
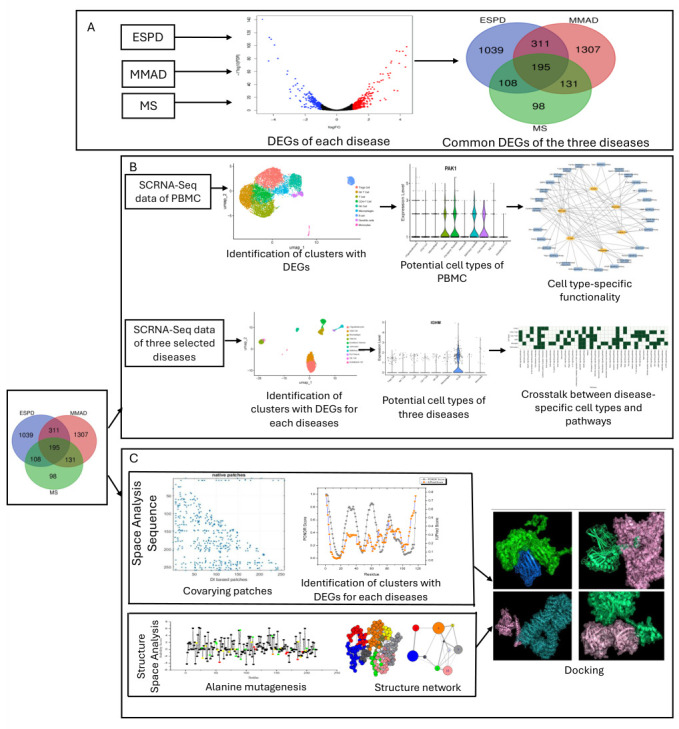
The detailed framework of the proposed work.

The second dataset utilized in this study comprises ∼10 k (scRNA-seq) profiles derived from peripheral blood mononuclear cells (PBMCs) obtained from healthy female donors between the ages of 25 and 30 [54]. We consider the raw count data to identify the cell types involved in the blood tissue. The healthy PBMC dataset was used primarily for immune cell-type annotation and marker localization rather than disease-state differential analysis. The flow chart of the proposed framework is shown in Figure 7A–C.

### 4.2. Processing and Cell-Type Identification

R software (4.6.0) is used to find out the differentially expressed proteins of each disease dataset. These expressed proteins in each disease sample are analyzed by using the limma package [55] in R. In this regard, Log2(fold change) − 1 ≤ (log _2_FC) ≥ 1 and a *p*-value ≤ 0.05 are used as the threshold criteria of the differentially expressed protein samples.

Raw count matrix processing was carried out using Seurat V3.0 [56]. Cell filtering was applied based on two criteria: cells exhibiting fewer than 200 or greater than 2500 unique feature counts were excluded, along with any cells in which mitochondrial read counts exceeded 5%. Following the removal of low-quality cells, data normalization was performed through the “LogNormalize” method, wherein raw counts were scaled by a factor of 10,000 and subsequently subjected to natural logarithm transformation. A K-Nearest Neighbor (KNN) graph was then constructed using principal components, upon which community detection was performed via the Louvain algorithm implemented through the “FindClusters” function. The resulting cell populations were visualized using Uniform Manifold Approximation and Projection (UMAP). To identify cluster-defining gene markers, the “FindMarkers” function was employed with the Wilcoxon Rank Sum test, applying a minimum expression percentage threshold of 0.25 across differentially expressed genes within each cluster.

### 4.3. Immune Cell-Specific Neuro-Marker Identification

From the differentially expressed autoantibody biomarkers of each disease, the common biomarkers are identified and shown through a Venn diagram. Biomarkers of all possible combinations of the diseases are compared with the clustered markers resulting from the single-cell analysis. Depending on the comparison, the autoimmune biomarkers are differentiated into diverse cell types. This represents the association of immune cell types in neuro diseases.

### 4.4. Pathway Crosstalk Network

The proteins common among two diseases, such as AD-PD, AD-MS and PD-MS, are considered to understand the activation/inhibition of the signaling pathways in the disease state. Moreover, another objective is also to identify the communication path among the immune cell types showing a higher impact on neuro disease progression. In this regard, the signaling pathways of the observed set of proteins are retrieved from three well-known publicly available databases: KEGG pathway [57], Wikipathway [58] and Reactome [59]. Among the extensive list of curated pathways, only signaling pathways are selected. This determined pathway list is further analyzed in detail. Moreover, we recognized the path through which the pathways activate/inhibit each other in a cell environment to maintain the proper balance. However, during this study, the chance of abnormalities that lead to the process of disease formation and progression is also highlighted.

Additionally, the marker associated with the selected signaling pathways is considered to understand the cell type associated with each pathway at a disease state. In this regard, we predefined a threshold, such as if 70% markers of a pathway show a connection with a particular cell type, we will acknowledge that pathway for the respective cell type.

### 4.5. Direct Coupling Analysis

Secondary correlation between non-interacting residues may arise from correlations between substitution patterns of the interacting ones. In order to investigate native contacts in a more specific way, direct couplings needed to be understood explicitly. A major shortcoming of the covariance study, i.e., the MI theory, was that it cannot disentangle direct correlations from indirect ones. Direct information (DI) is one such case on MI (Mutual Information), which aims at disentangling direct interactions from indirect ones. Based on DI scoring (Supplementary Table S2), direct coupling analysis (DCA) was performed. Therefore, from DCA, a lucid view of DI (Equation (1)), i.e., how directly the selected coincide sites are coupled with each other, was obtained.

(1) $$DI_{i,j}=\sum AB\;P_{i,j}^{\left(dir\right)}\;\left(A,B\right)\;ln\frac{P_{i,j}^{\left(dir\right)}}{P_{i}\left(A\right)P_{j}\left(B\right)}$$

Here, $P_{i}j(\left(dir\right))$ represents reweighted frequency counts to introduce two residues for DI, where P(A,B) is considered as joint probability, and P(A) and P(B) are individual probabilities. $P_{i}\left(A\right)$ and $P_{j}\left(B\right)$ are for amino acid type A at ith position and similarly B at jth position.

### 4.6. Model Building

Here, sequences of P55889 and Q13188 were retrieved from UniProt and their models were generated using I-TASSER [60,61]. Coordinate information of the partner proteins were obtained from the Protein Data Bank (PDB). The generated models and the selected PDBs were further subjected to structure network analysis.

### 4.7. Structure Network Analysis

In order to explore the internal organization and inter-dependency of the amino acid residues in terms of pairwise interactions in the model structures, structure network analysis was deployed. The interaction established between the elements of the networks, i.e., the residues depending on the interaction energy and/or spatial distance, were represented through edges and nodes. Based on the interaction strength, the sides were drawn between the two amino acid nodes. The equation is represented below.

(2) $$F_{ij}=\left[\frac{x_{ij}}{\sqrt{\left(X_{i}*X_{j}\right)}}\right]*100\geq F_{c}$$

where $F_{c}$ is the threshold of interaction strength and the default value is 4%. Here, $x_{ij}$ is the number of side chain atom pairs of residues *i* and *j*. $X_{i}$ and $X_{j}$ are the normalization factors for residue types *i* and *j* [62,63].

### 4.8. Stability Score Calculation

Additionally, the stability score of the amino acid present in a protein was calculated by alascan mutagenesis technique. Similarly, steric clashes of the amino acid were also performed. This was one of the artifacts prevalent in low-resolution structures and homology models. Steric clashes arised due to the unnatural overlap of any two non-bonding atoms in a protein structure. Usually, removal of severe steric clashes in some structures is challenging since many existing refinement programs did not accept structures with severe steric clashes. Due to the non-bulky, chemically inert methyl functional group that nevertheless mimics the secondary structure preferences, alanine is used to check the stability.

### 4.9. Expression Validation Using Independent Public Datasets

To independently validate the selected seed markers (kinases and immunoglobulins), expression data from three publicly available datasets—GSE1297 [30], GSE165082 [31], and GSE108000 [32]—were retrieved and processed. For each dataset, expression levels of the selected markers were compared between control and diseased samples using appropriate statistical tests. Differential expression was assessed and visualized using box plots. Markers reaching statistical significance were considered validated.

### 4.10. Molecular Docking Validation

To provide a mechanistic understanding of the protein–protein association that has manifested from our previous analysis, we deployed the computational strategy of protein–protein docking [64]. Models built from I-TASSER along with the selected coordinate structures were subjected to the docking study and tools from ClusPro were used [65]. In this rigid body docking, the angular step size for rotational sampling of ligand orientations was set to about 5° in terms of Eular angles. Energy minimization followed by root-mean-square deviation (RMSD)-based clustering was performed for accurate and near-native conformational sampling and refinement of the complex structure. In each case, docked conformation with the lowest energy value was considered. Along with the energy value, conformations with the highest number of members involved were also prioritized in order to study a precise complex encounter and also to support the hypothesis that the interaction between two candidate structures took place in the neighborhood of the native state.

## Data Availability

The original contributions presented in this study are included in the article. Further inquiries can be directed to the corresponding author(s).

## Supplementary Materials

The following supporting information can be downloaded at https://www.mdpi.com/article/10.3390/ijms27125627/s1.
