# Supplementary material for: IMMUND: A Diagnostic and Therapeutic Pipeline to Uncover the Convergence in Functional Perturbation at Early Stages of Neurodegenerative Diseases and Multiple Sclerosis Based on Protein Markers
Source: Int J Mol Sci. 2026 Jun 22;27(12):5627. doi: 10.3390/ijms27125627 (PMC13299175; doi:10.3390/ijms27125627)
Supplement: Supplementary file 1 [file ijms-27-05627-s001.zip › Supplementary Figures.pptx]

## Slide 1
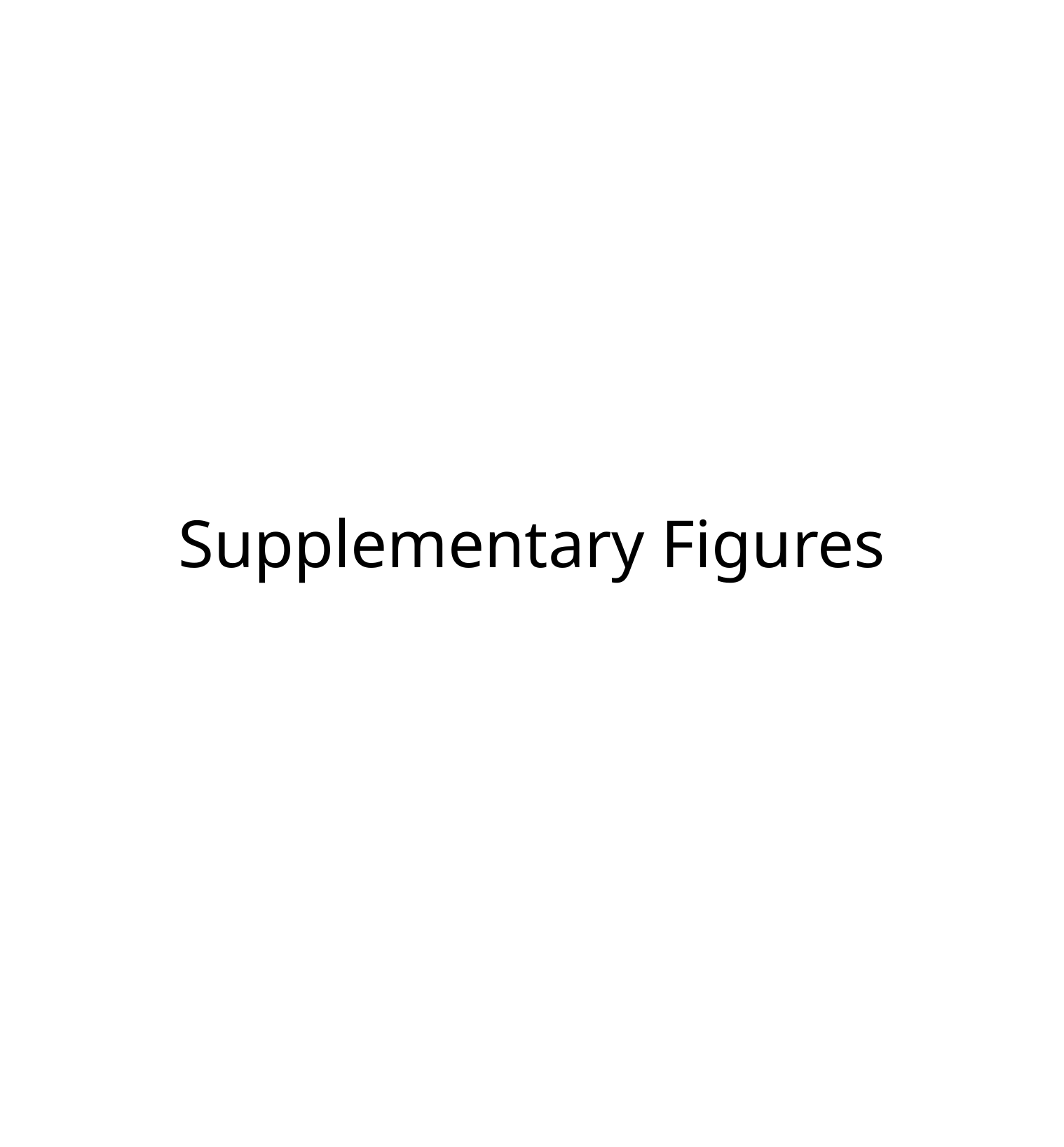

# Supplementary Figures

## Slide 2
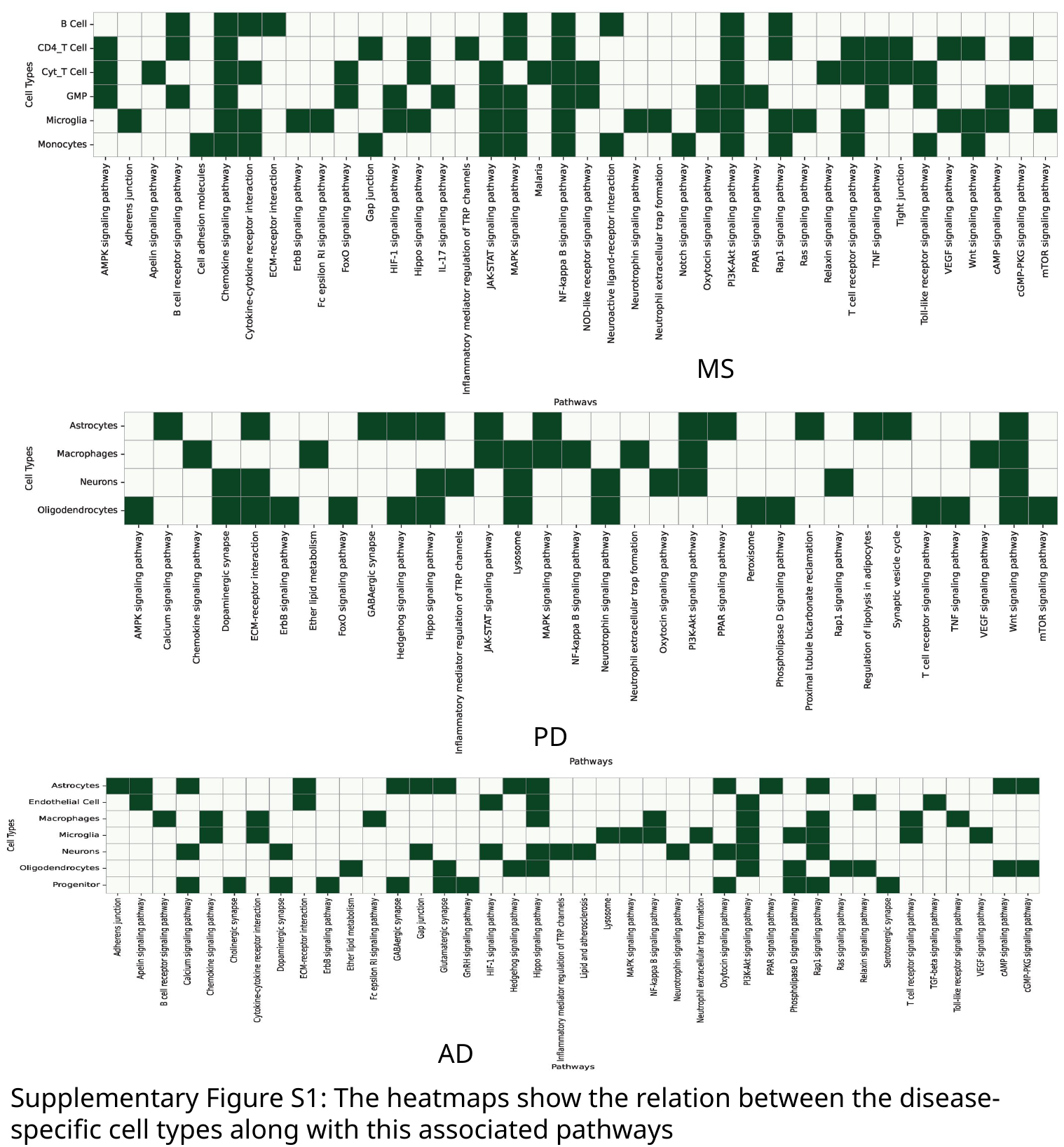

MS
PD
AD
Supplementary Figure S1: The heatmaps show the relation between the disease-specific cell types along with this associated pathways

## Slide 3
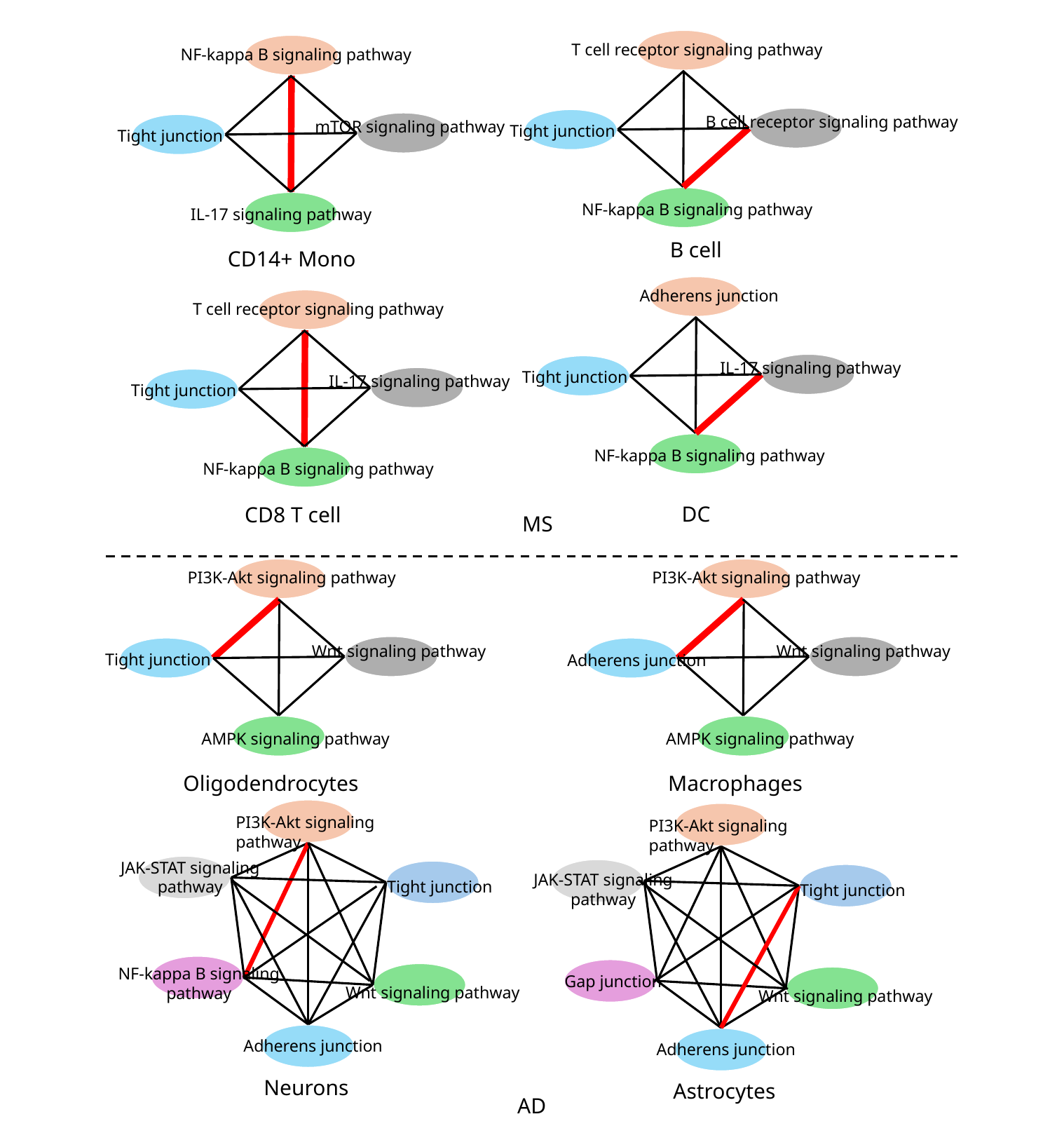

T cell receptor signaling pathway
B cell receptor signaling pathway
Tight junction
NF-kappa B signaling pathway
NF-kappa B signaling pathway
mTOR signaling pathway
Tight junction
IL-17 signaling pathway
B cell
CD14+ Mono
Adherens junction
T cell receptor signaling pathway
IL-17 signaling pathway
Tight junction
NF-kappa B signaling pathway
IL-17 signaling pathway
Tight junction
NF-kappa B signaling pathway
DC
CD8 T cell
MS
PI3K-Akt signaling pathway
Wnt signaling pathway
Tight junction
AMPK signaling pathway
PI3K-Akt signaling pathway
Wnt signaling pathway
Adherens junction
AMPK signaling pathway
Oligodendrocytes
Macrophages
PI3K-Akt signaling pathway
PI3K-Akt signaling pathway
JAK-STAT signaling pathway
JAK-STAT signaling pathway
Tight junction
Tight junction
NF-kappa B signaling pathway
Gap junction
Wnt signaling pathway
Wnt signaling pathway
Adherens junction
Adherens junction
Neurons
Astrocytes
AD

## Slide 4
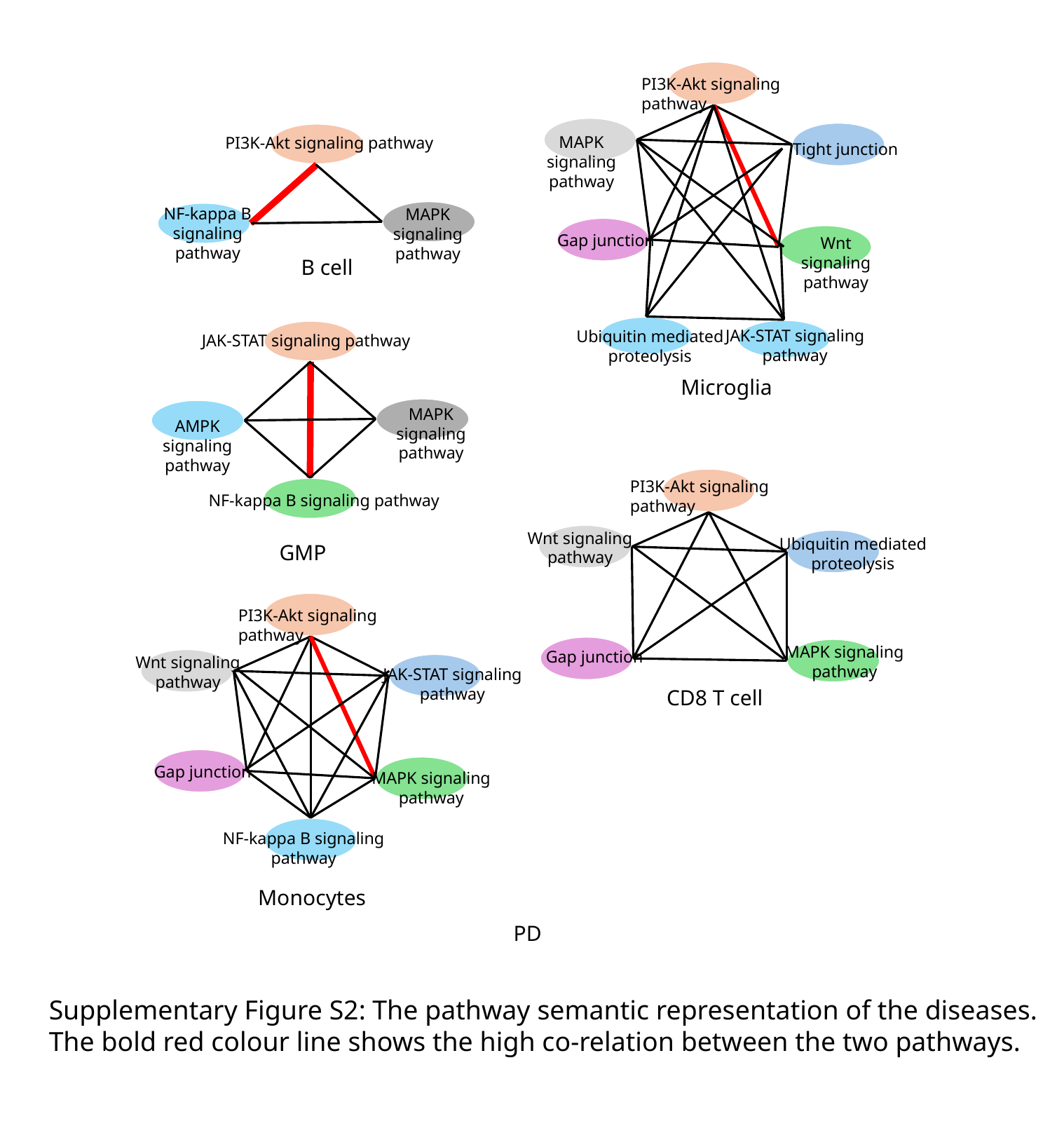

PI3K-Akt signaling pathway
MAPK signaling pathway
Tight junction
Gap junction
Wnt signaling pathway
JAK-STAT signaling pathway
Ubiquitin mediated proteolysis
PI3K-Akt signaling pathway
NF-kappa B signaling pathway
MAPK signaling pathway
B cell
JAK-STAT signaling pathway
MAPK signaling pathway
AMPK signaling pathway
NF-kappa B signaling pathway
Microglia
PI3K-Akt signaling pathway
Wnt signaling pathway
Ubiquitin mediated proteolysis
GMP
PI3K-Akt signaling pathway
Wnt signaling pathway
JAK-STAT signaling pathway
Gap junction
MAPK signaling pathway
NF-kappa B signaling pathway
MAPK signaling pathway
Gap junction
CD8 T cell
Monocytes
PD
Supplementary Figure S2: The pathway semantic representation of the diseases. The bold red colour line shows the high co-relation between the two pathways.

## Slide 5
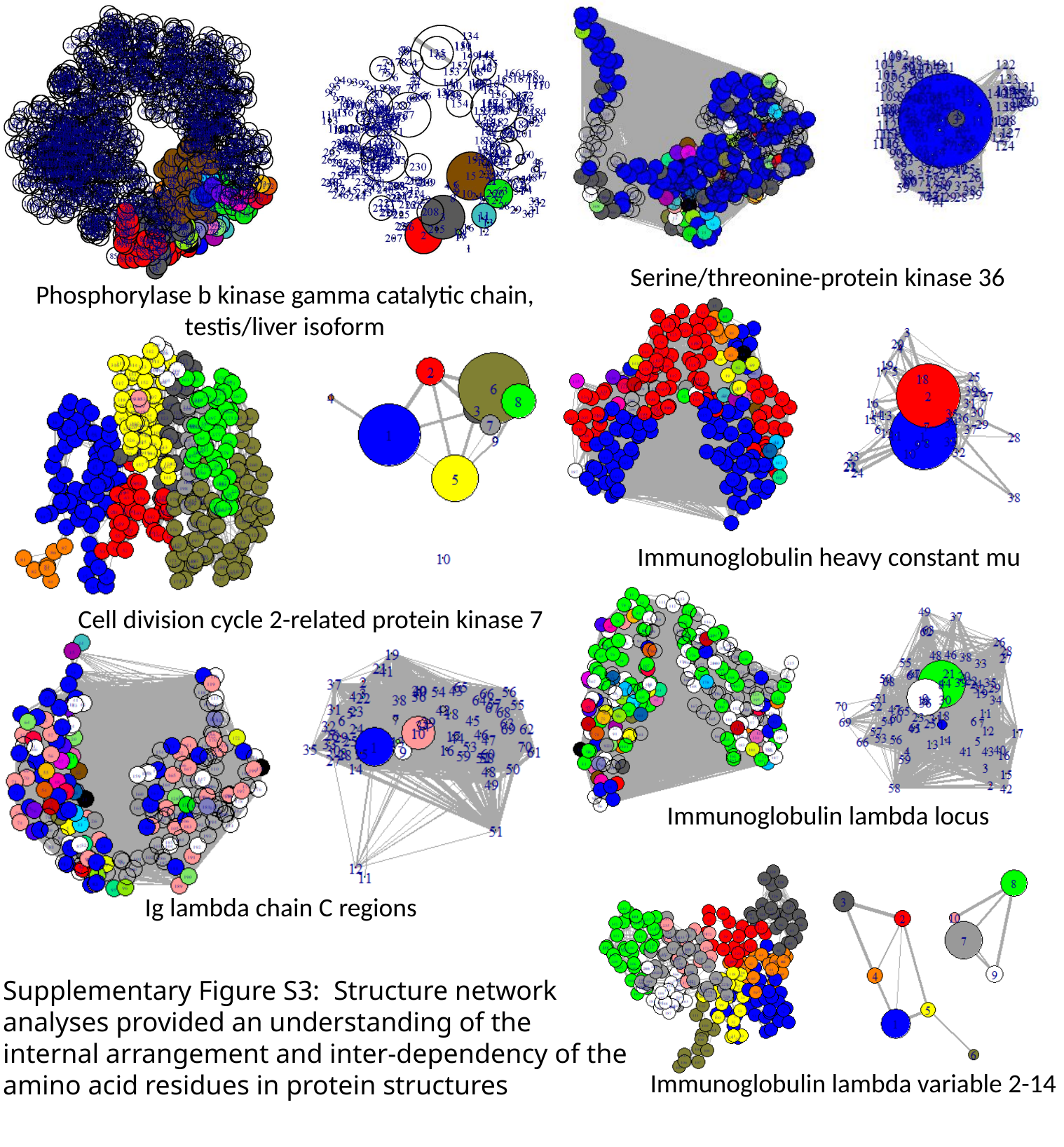

Serine/threonine-protein kinase 36
Phosphorylase b kinase gamma catalytic chain, testis/liver isoform
Immunoglobulin heavy constant mu
Cell division cycle 2-related protein kinase 7
Immunoglobulin lambda locus
Ig lambda chain C regions
Supplementary Figure S3: Structure network analyses provided an understanding of the internal arrangement and inter-dependency of the amino acid residues in protein structures
Immunoglobulin lambda variable 2-14

## Slide 6
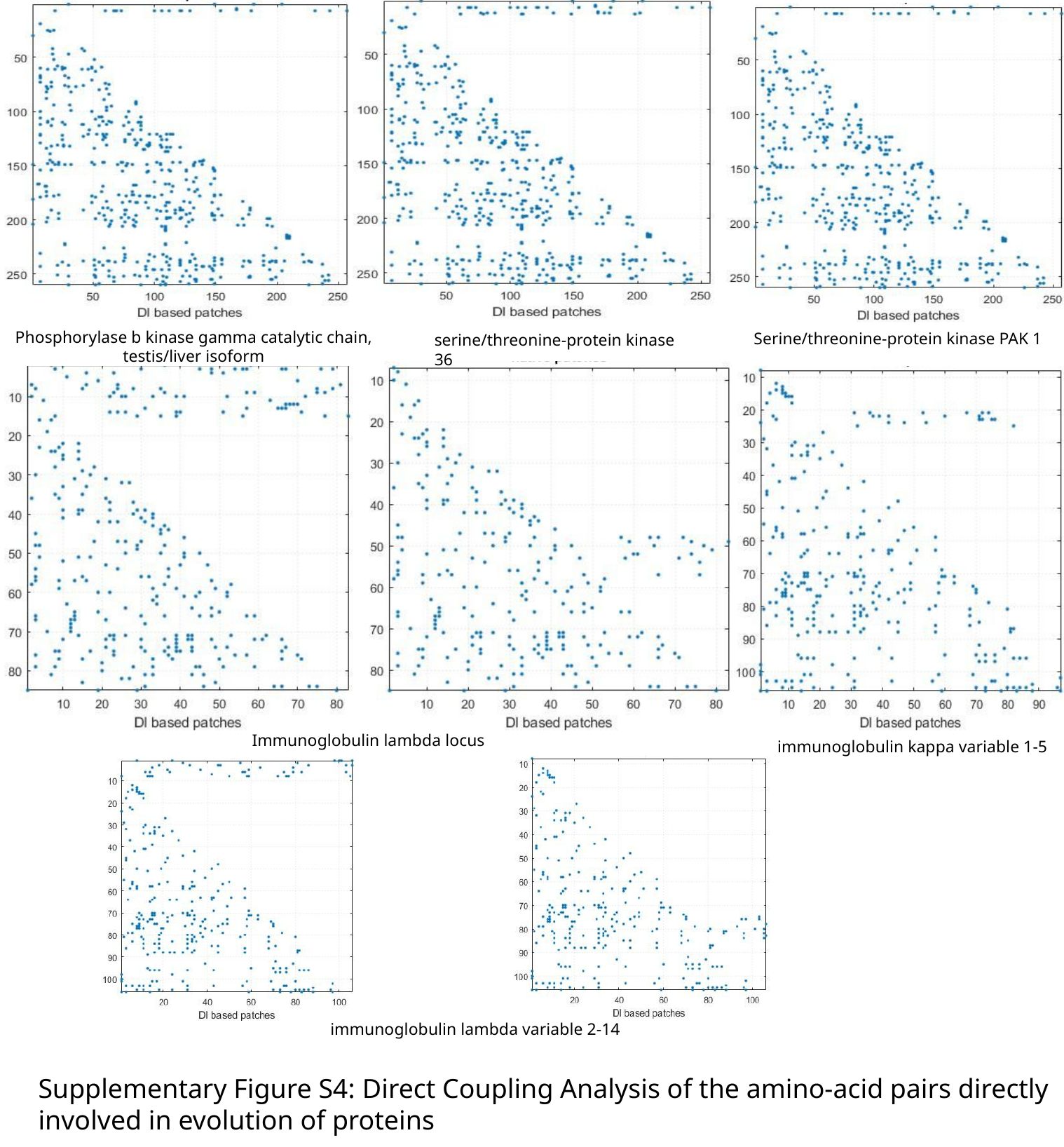

Phosphorylase b kinase gamma catalytic chain, testis/liver isoform
Serine/threonine-protein kinase PAK 1
serine/threonine-protein kinase 36
Immunoglobulin lambda locus
immunoglobulin kappa variable 1-5
immunoglobulin lambda variable 2-14
Supplementary Figure S4: Direct Coupling Analysis of the amino-acid pairs directly involved in evolution of proteins

## Slide 7
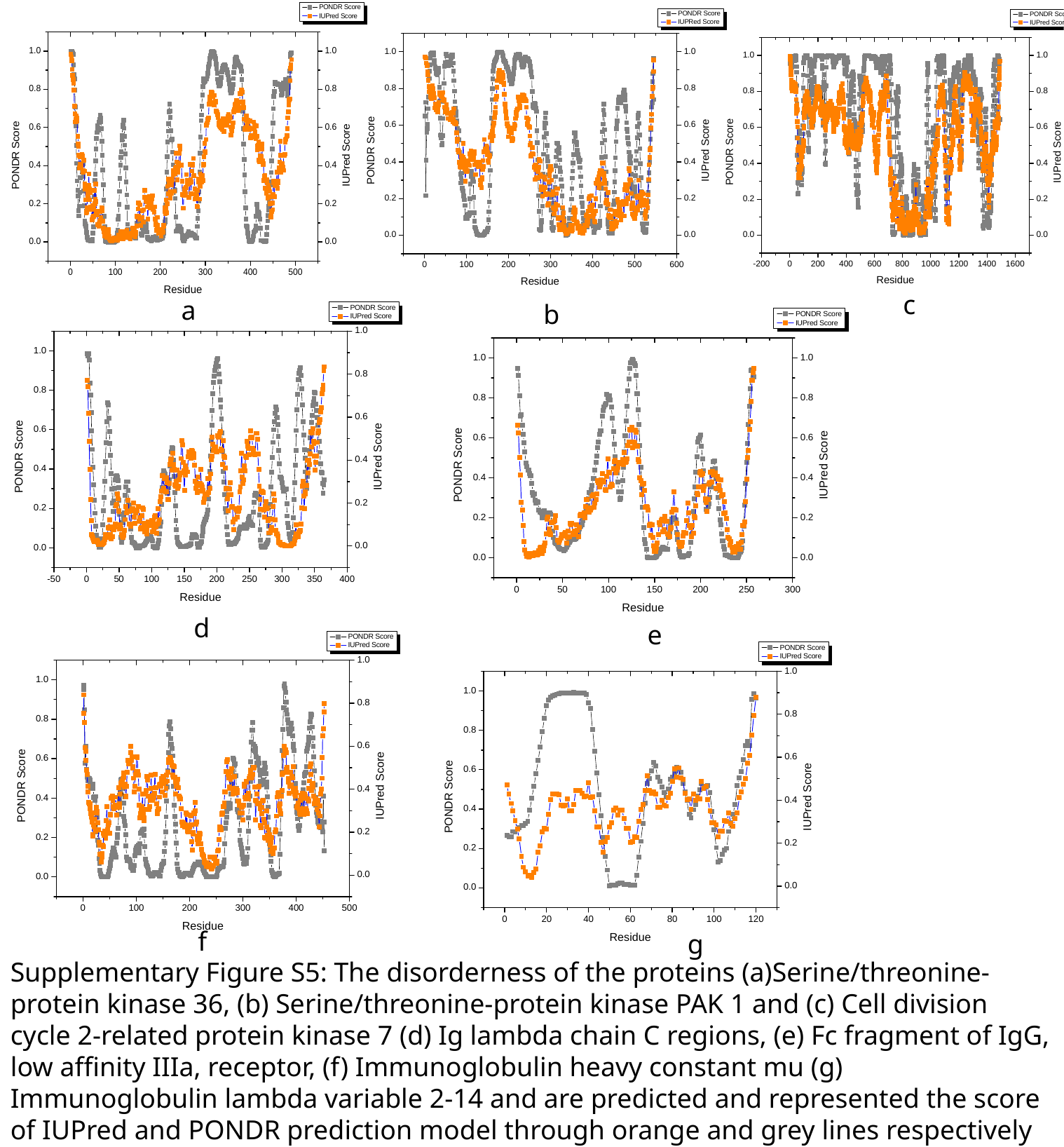

c
a
b
d
e
f
g
Supplementary Figure S5: The disorderness of the proteins (a)Serine/threonine-protein kinase 36, (b) Serine/threonine-protein kinase PAK 1 and (c) Cell division cycle 2-related protein kinase 7 (d) Ig lambda chain C regions, (e) Fc fragment of IgG, low affinity IIIa, receptor, (f) Immunoglobulin heavy constant mu (g) Immunoglobulin lambda variable 2-14 and are predicted and represented the score of IUPred and PONDR prediction model through orange and grey lines respectively

## Slide 8
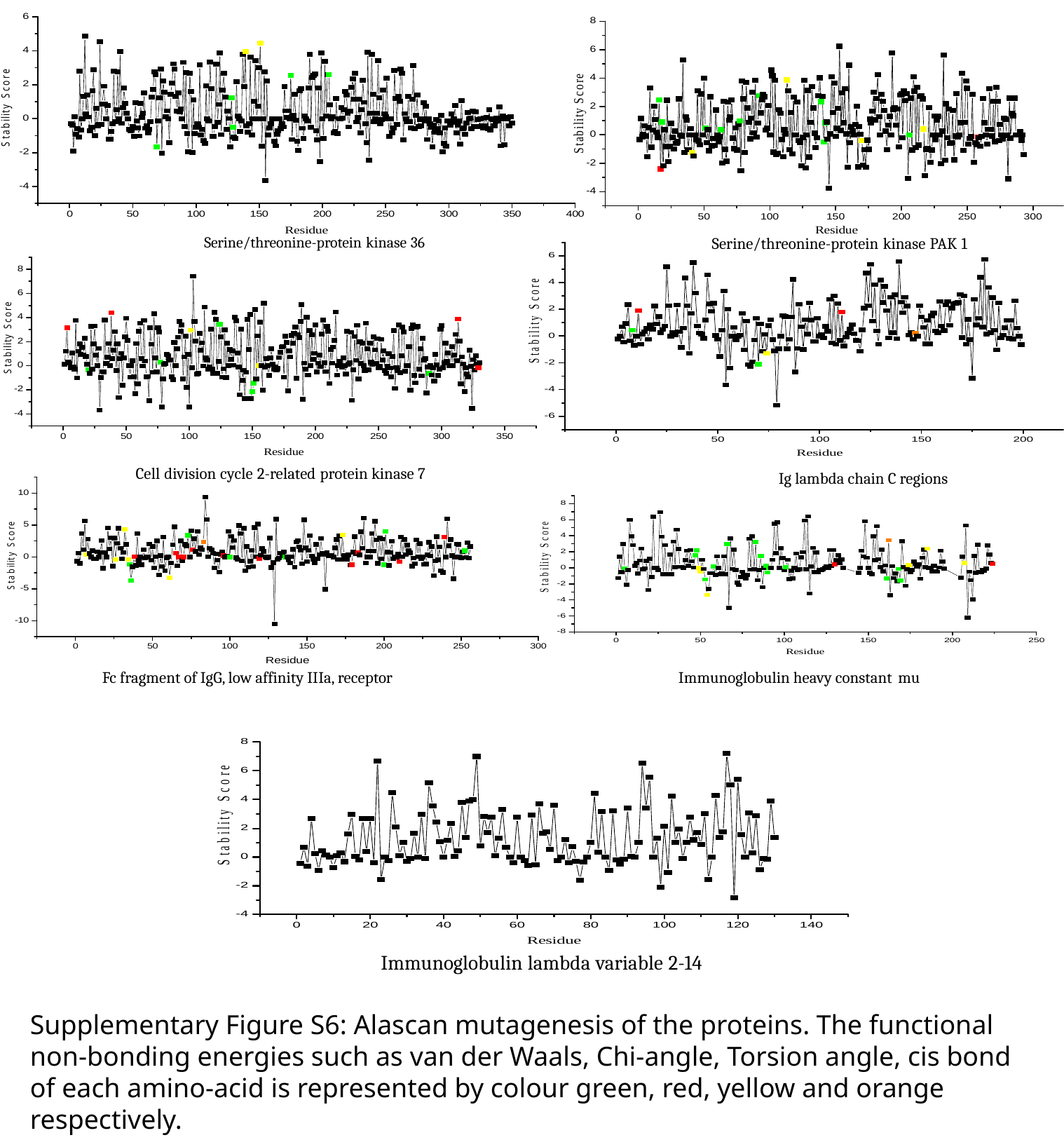

Serine/threonine-protein kinase 36
Serine/threonine-protein kinase PAK 1
Cell division cycle 2-related protein kinase 7
Ig lambda chain C regions
Fc fragment of IgG, low affinity IIIa, receptor
Immunoglobulin heavy constant mu
Immunoglobulin lambda variable 2-14
Supplementary Figure S6: Alascan mutagenesis of the proteins. The functional non-bonding energies such as van der Waals, Chi-angle, Torsion angle, cis bond of each amino-acid is represented by colour green, red, yellow and orange respectively.

## Slide 9
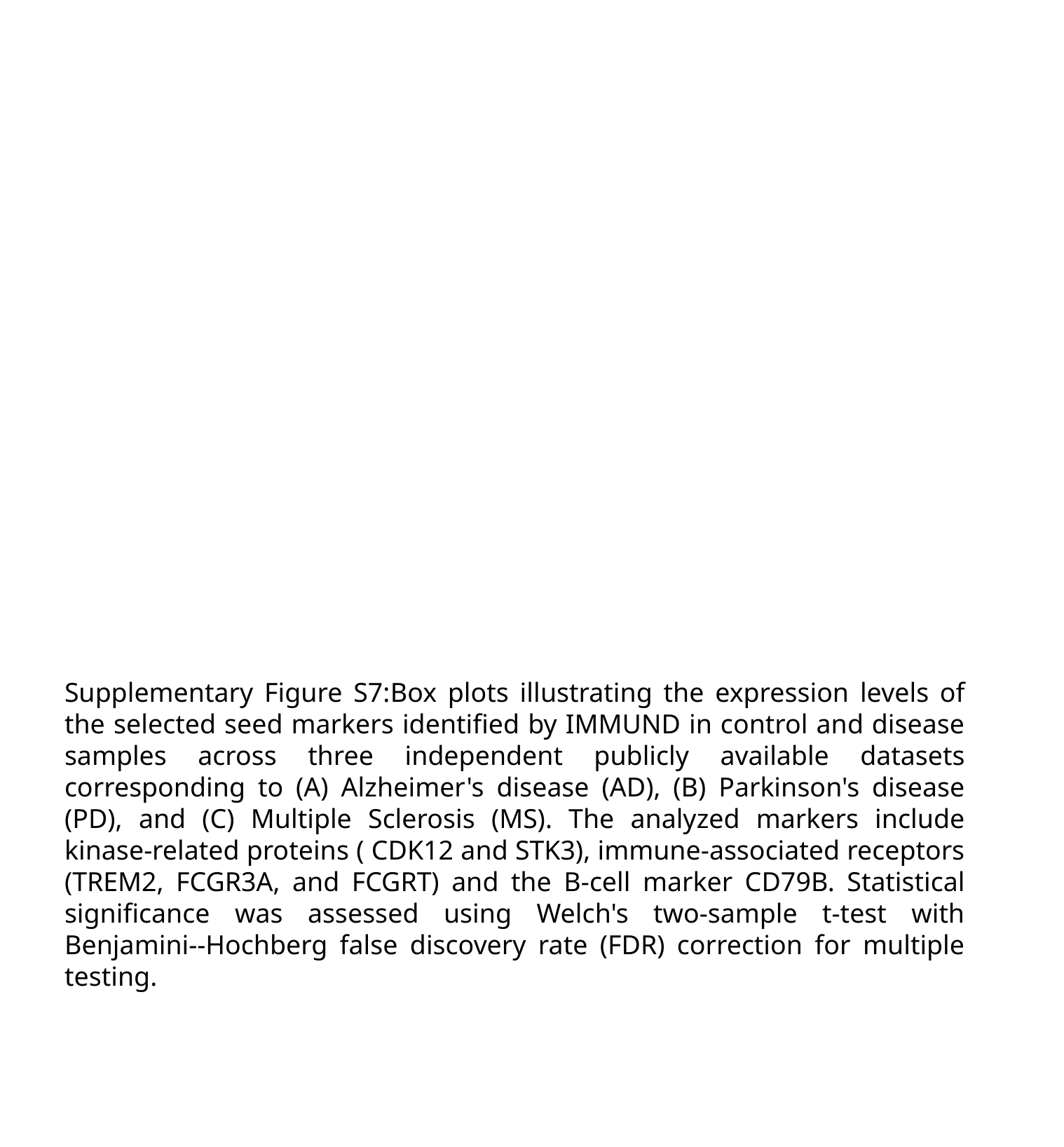

Supplementary Figure S7:Box plots illustrating the expression levels of the selected seed markers identified by IMMUND in control and disease samples across three independent publicly available datasets corresponding to (A) Alzheimer's disease (AD), (B) Parkinson's disease (PD), and (C) Multiple Sclerosis (MS). The analyzed markers include kinase-related proteins ( CDK12 and STK3), immune-associated receptors (TREM2, FCGR3A, and FCGRT) and the B-cell marker CD79B. Statistical significance was assessed using Welch's two-sample t-test with Benjamini--Hochberg false discovery rate (FDR) correction for multiple testing.
